# Are smart glasses feasible for dispatch prehospital assistance during on-boat cardiac arrest? A pilot simulation study with fishermen

**DOI:** 10.1007/s11739-023-03251-6

**Published:** 2023-04-04

**Authors:** Roberto Barcala-Furelos, Silvia Aranda-García, Martín Otero-Agra, Felipe Fernández-Méndez, Alejandra Alonso-Calvete, Santiago Martínez-Isasi, Robert Greif, Antonio Rodríguez-Núñez

**Affiliations:** 1 REMOSS Research Group, Faculty of Education and Sport Sciences, Universidade de Vigo, Pontevedra, Spain; 2 CLINURSID Research Group, School of Nursing, Universidade de Santiago de Compostela, Santiago de Compostela, Spain; 3 Life Support and Medical Simulation Research Group (SICRUS), Health Research Institute of Santiago de Compostela (IDIS), Santiago de Compostela, Spain; 4 GRAFAIS Research Group, Institut Nacional d’Educació Física de Catalunya (INEFC), Universitat de Barcelona, Av. De l’Estadi 22, 08038 Barcelona, Spain; 5 School of Nursing from Pontevedra, Universidade de Vigo, Pontevedra, Spain; 6 Faculty of Physiotherapy, Universidade de Vigo, Pontevedra, Spain; 7 Faculty of Nursing, Universidade de Santiago de Compostela, Santiago de Compostela, Spain; 8 University of Bern, Bern, Switzerland; 9 School of Medicine, Sigmund Freud University Vienna, Vienna, Austria; 10 Paediatric Critical, Intermediate and Palliative Care Section, Santiago de Compostela’s University Hospital, Santiago de Compostela, Spain

**Keywords:** Smart glasses, Video dispatch, Out-of-hospital cardiac arrest, Bystander, Cardiopulmonary resuscitation

## Abstract

The aim of the study was to explore feasibility of basic life support (BLS) guided through smart glasses (SGs) when assisting fishermen bystanders. Twelve participants assisted a simulated out-of-hospital cardiac arrest on a fishing boat assisted by the dispatcher through the SGs. The SGs were connected to make video calls. Feasibility was assessed whether or not they needed help from the dispatcher. BLS-AED steps, time to first shock/compression, and CPR’s quality (hands-only) during 2 consecutive minutes (1st minute without dispatcher feedback, 2nd with dispatcher feedback) were analyzed. Reliability was analyzed by comparing the assessment of variables performed by the dispatcher through SGs with those registered by an on-scene instructor. Assistance through SGs was needed in 72% of the BLS steps, which enabled all participants to perform the ABC approach and use AED correctly. Feasibility was proven that dispatcher’s feedback through SGs helped to improve bystanders’ performance, as after dispatcher gave feedback via SGs, only 3% of skills were incorrect. Comparison of on-scene instructor vs. SGs assessment by dispatcher differ in 8% of the analyzed skills: greatest difference in the "incorrect hand position during CPR" (on-scene: 33% vs. dispatcher: 0%). When comparing the 1st minute with 2nd minute, there were only significant differences in the percentage of compressions with correct depth (1st:48 ± 42%, 2nd:70 ± 31, *p* = 0.02). Using SGs in aquatic settings is feasible and improves BLS. CPR quality markers were similar with and without SG. These devices have great potential for communication between dispatchers and laypersons but need more development to be used in real emergencies.

**Supplementary Information:**

The online version contains supplementary material available at 10.1007/s11739-023-03251-6.

## Introduction

The 2021 European Resuscitation Council (ERC) Guidelines suggest that emergency medical services (EMS) should consider the use of technology such as video communication to communicate with bystanders and provide dispatcher-assisted cardiopulmonary resuscitation (CPR) [1]. Smartphones are currently the most frequently used devices for video calls. However, the future of video communication could be the head-worn devices (HWD), which are portable, hands-free gadgets with a small optic in front of the eyes. These innovative devices can help in different areas related to physical and cognitive workload or in task complexity [2]. Smart glasses (SGs) are considered a HWD and allow communication between a receiver (dispatcher) and the user in real time. This communication can favor that there is better performance in emergencies in which the bystander needs the help of the dispatcher [3]. The performance of SGs has increased in terms of a longer battery life, less weight, greater comfort, enhanced hardware and software improvements with new cutting-edge communication apps [2].

In the out-of-hospital emergency field, a potential application which does not yet have usability analysis is the video-streaming dispatch with SGs used by bystanders in remote areas like the ocean coast. Out-of-hospital cardiac arrest (OHCA) in peculiar settings such as water areas is described in the ERC guidelines as a special resuscitation situation [4].

The target population for this study were fishermen who have worked in high-risk situations for many years. Fishing is a high-risk profession in which one-fifth of fishermen had been involved in a medical emergency at sea that required them to be evacuated to shore for immediate treatment [5]. 1.3% of very serious accidents during fishing are heart attacks, and an additional 38.7% are situations that can cause cardiorespiratory arrests such as drowning or hypothermia [6]. They are usually located far away from specialized medical help, with an extremely limited number of witnesses nearby [7]. This means that medical emergencies in aquatic settings are, inevitably potentially recurring situations, bearing in mind such an uncontrolled and remote environment.

Under these conditions, the hypothesis of this feasibility study was that basic life support (BLS) guided by an emergency dispatcher through SGs would improve and assist bystander's decision making and the performance of correct BLS steps as primary outcome. Furthermore, secondary outcomes were the reliability and accuracy of the emergency dispatcher's assessment, which would be comparable to an on-scene dispatcher (the gold standard), and CPR-quality markers. Therefore, the aim of this pilot simulation study was to analyze the sequence of the ABC approach, the automated external defibrillator (AED) use, and the correct steps of the CPR skills performed by fishermen who are assisted using the SGs innovative tool.

## Methods

### Design

A descriptive and comparative design was used to test the feasibility and reliability of SGs during video assistance in a simulated OHCA undertaken by fishermen sailing in a small fishing boat.

### Participants

**Fig. 1:**
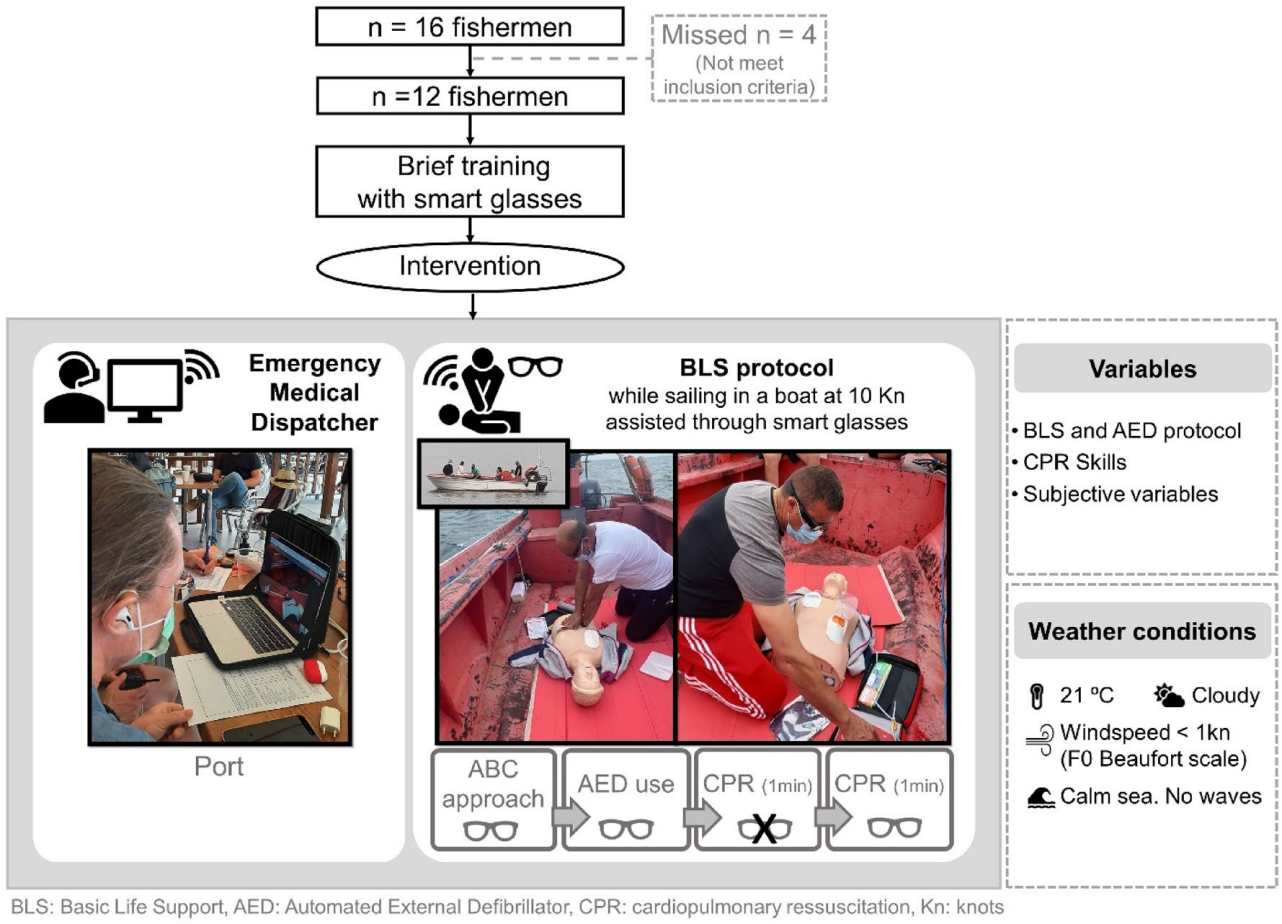
Flow chart outlining the design and procedures

A total of 16 coastal fishermen were invited to participate in this study. The inclusion criteria were to be professional fishermen with at least 10 years of experience (to ensure fully familiar with the boat and with performing tasks while sailing) who had not undergone BLS training in the previous 6 months. Finally, 12 fishermen (100% male) were included. Four were excluded because they had received BLS training within the last 6 months. The mean age of participants was 46 ± 4 years (95% confidence interval [CI] 43–48) (Fig. 1). Before starting the study, informed consent was requested from all participants. The study was approved by the Ethics Committee for Clinical Research of the Catalan Sports Council (022/CEICGC/2021).

### Very brief training on smart glasses and connection details

**Fig. 2:**
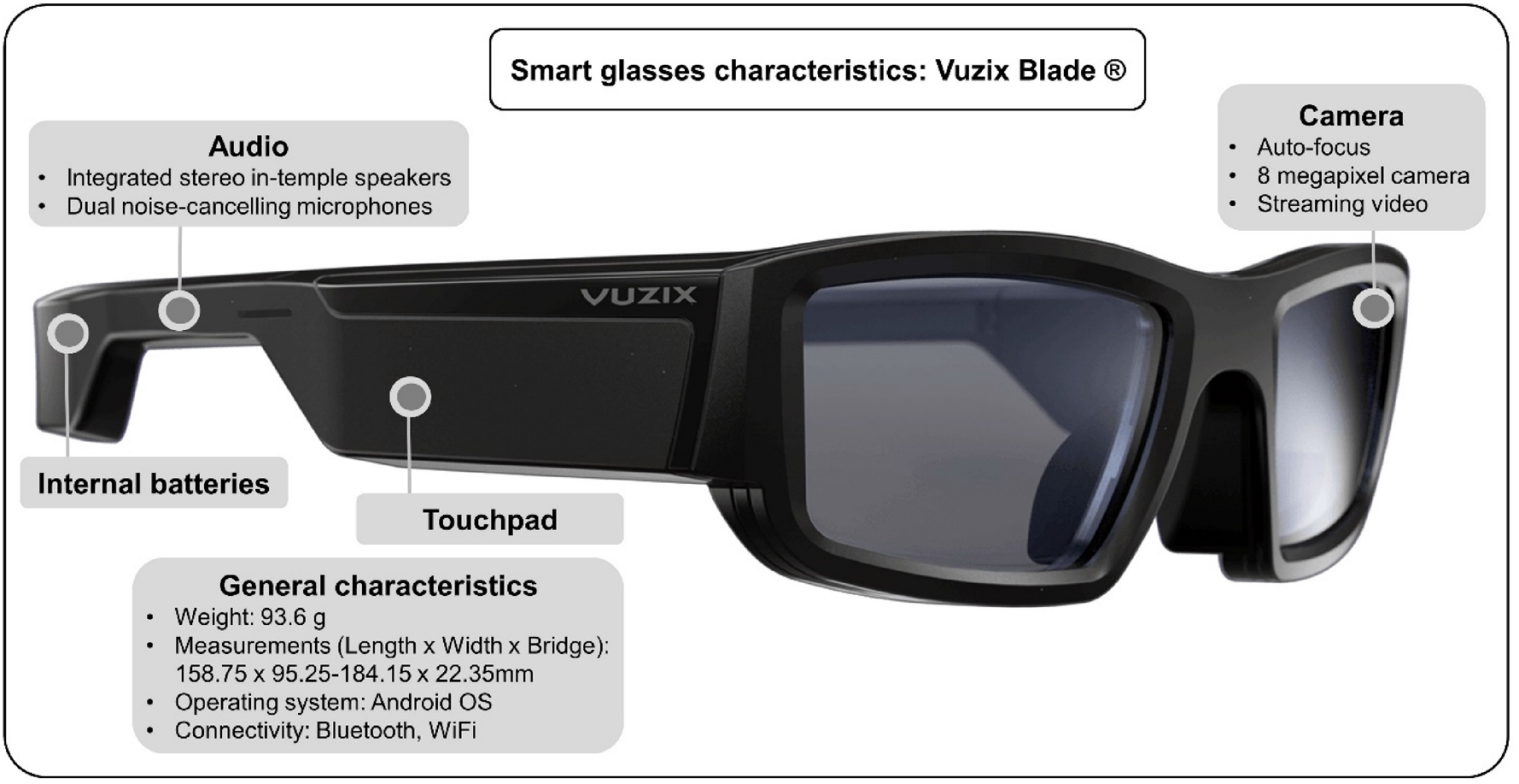
Smart glasses characteristics

The first phase of the pilot study consisted of the fishermen's familiarization with the use of SGs. The training was conducted on board of a boat that was moored in the harbor with the engine turned off. Each participant put on the SGs for 5 min and had a brief conversation with the emergency dispatcher who was located at a facility near the harbor. The connection to the SGs (Vuzix Blade AR, United States) was made via a 4G wireless network, previously configured using the VRA Mobile App (Vuzix, United States). The SG’s characteristics can be seen in Fig. 2.

### Trial environment

The connection was established by navigating at 10kn at half a nautical mile from land. The fishing boat was 6 m long and its beam measured 3 m. The crew consisted of a skipper at the helm, a fisherman (the bystander), and two people from the research team (a communication technician and a BLS instructor). Data collection was performed on August 20, 2021, between 16:00 and 20:00 in the port of Rianxo (Spain), located on the Atlantic coast at GPS position: 42°38′49″–8°49′30″. The weather conditions were: 21 °C, cloudy, with a wind speed < 1 kt (F0 on the Beaufort scale), in a calm sea with no waves.

### Clinical simulation and variables

*Step 1* Cardiac arrest on boat.

The on-boat instructor presented the following clinical simulation individually to each participant:

"A fisherman from the crew collapses on the boat while performing some physical exertion. The victim is on the ground and is simulated by a manikin. The boat is also equipped with an AED. You have the SGs on and they are connected to a control center" (see supplemental video).

*Step 2* ABC approach and AED response.

The bystander had to initiate victim assistance when the boat was cruising at 10kn and when instructed to do so by the researcher.

Analysis variables consisted of the following BLS steps: 1. check response, 2. open victim´s airway, 3. check breathing, 4. bring in an AED, 5. place the AED pads properly, 6. deliver a shock, 7. perform chest compressions (CC) after AED shock, and 8. correct hand placement during CC. Feasibility was assessed dichotomously as YES (i.e., skill performed correctly by the witness without the need to dispatch) or NO (i.e., omission of the step or performed incorrectly/not effectively). Dispatcher assistance was provided through SGs if the fisherman did NOT perform the step, did not perform the step in the correct order or performed it incorrectly. Time (in seconds) from the start of the procedure to the first shock and the first cardiac compression was analyzed quantitatively.

Reliability was analyzed by comparing the assessment of each variable performed by the dispatcher through the SGs with an on-scene instructor in the boat, who recorded the same eight variables. The instructor did not interact with the participants at any time.

*Step 3* CPR skills with SGs dispatch.

After step 2, the bystanders initiated hands-only CPR for 2 min. During the 1st minute, they received no communication from the dispatcher and during the 2nd minute, they received continuous feedback from the dispatcher; min 1 (no dispatcher feedback) vs. min 2 (with dispatcher feedback).

The evaluated CPR-quality markers were: (a) CC with a correct rate as a percentage (CC-RA %), (b) CC with correct depth as a percentage (CC-D %), (c) CC with full chest release as a percentage (CC-RE %), and (d) CC with correct hand position as a percentage (CC-HP %). The reference values were those indicated for CC by the ERC guidelines in 2021 [1]; at a rate of 100–120 cc/min and depth of 50–60 mm with full chest recoil and hand placement in the middle of the chest. Skill recording was obtained using the APP CPR instructor (Laerdal, Norway), which was connected to the Little Anne QCPR manikin (Laerdal, Norway).

### Data analysis

Statistical analysis was performed with IBM SPSS Statistics for Windows software, version 20.0 (Armonk, NY: IBM Corp). Categorical variables were described through absolute and relative frequencies. Continuous variables were described through measures of central tendency (mean), dispersion (standard deviation) and confidence estimators (95% confidence intervals). Means comparisons were performed using Student's *t* test for variables that met the criteria of normality and the Wilcoxon rank sum test was used for variables that did not meet the criteria of normality. For comparisons that presented statistically significant differences, the effect size was calculated using the Rosenthal test and had the following classification: trivial (< 0.2); small (0.2–0.5); moderate (0.5–0.8); large (0.8–1.3); very large (> 1.3).

## Results

All 12 fishermen were able to complete the study by performing the ABC approach and using AED sequentially and correctly, but this was always achieved with the help of the dispatcher’s feedback through the SGs. The result analysis disaggregates: (a) the steps of the BLS protocol that the lay person (fisherman) omitted or performed incorrectly and what should have been indicated or corrected by the dispatcher through the SGs, (b) the reliability analysis of the SGs comparing the dispatcher's final evaluation with that of an on-scene instructor, (c) CRP-quality markers, and (d) time to first defibrillation and time to initiation of CC.

**Fig. 3:**
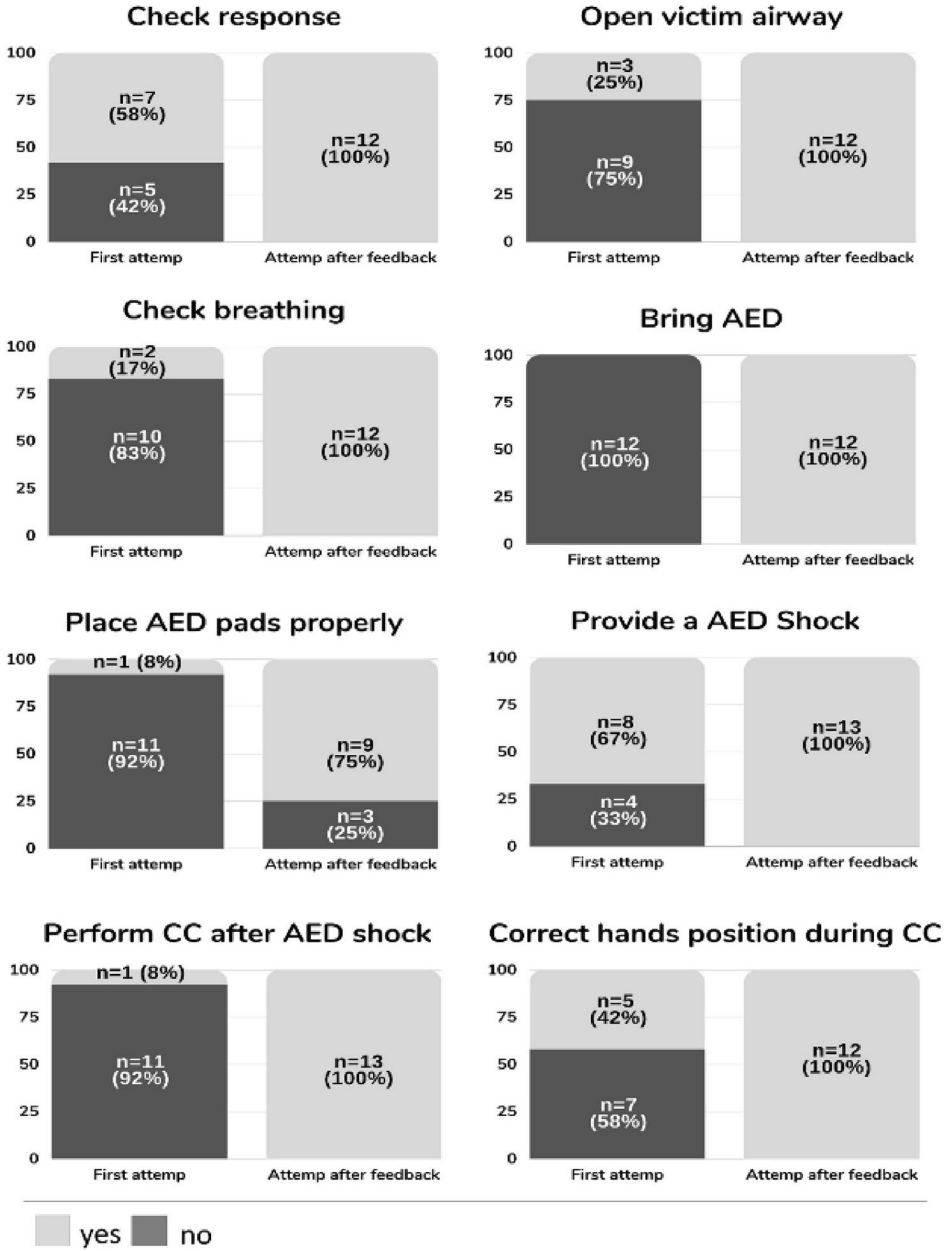
Percentage of correct steps in ABC approach before and after dispatcher assistance through smart glasses

**Fig. 4:**
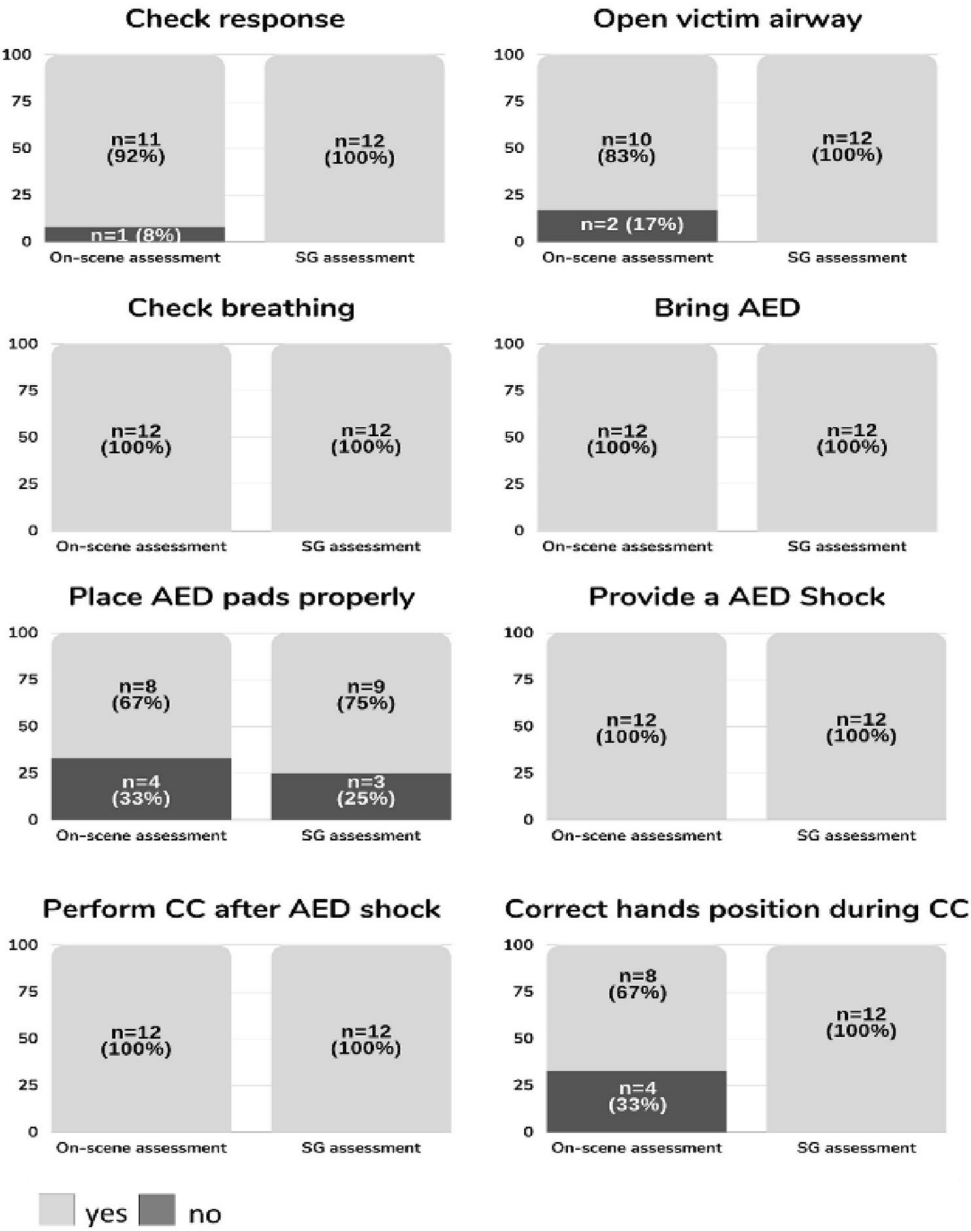
On-scene instructor vs. dispatcher evaluation comparison through smart glasses

- Feasibility results: the dispatcher had to give feedback to all participants (100%). Out of a total of 96 skills that were assessed, assistance was given in 65 of the steps, which represented 72% of the interventions. The skills that received the most instructions were those related to the AED use. After dispatch through the SGs, only 3% of skills were not completed at the dispatcher's discretion (Fig. 3).
- Reliability results: comparison of the on-scene instructor`s assessment vs. the SGs assessment by dispatcher differed in 8% of the skills analyzed. The largest difference was found in the variable "correct hand position during CC", which the dispatcher considered correct in 100% of the participants, while the on-scene instructor indicated that 33% were neither placing their hands in the center of the chest nor in accordance with the recommendations for resuscitation (Fig. 4).
- CPR-quality markers: the mean depth of compressions during the 2 min of resuscitation was 54 ± 11 mm (95% CI 47–61). We only found a significant difference in the variable CC depth (1st minute: 48 ± 42%, 2nd minute: 70 ± 31%, *p* = 0.02, EN = 0.48) (Table 1) comparing the 1st minute (without feedback dispatcher) and the 2nd minute (with dispatcher feedback through SGs). This represents 22% in favor of the 2nd minute with telecare using the SGs. The rest of the CPR variables remained unchanged between 1st and 2nd minutes.
- The time from the start of the intervention to defibrillation was 162 ± 26 s, and 14 ± 6 s from defibrillation to the start of CPR.

**Table 1:** Results of CPR skills by minute

	Without assistance with SGs (1st minute)		Video-assisted with SGs (2nd minute)		*p* value [effect size]
	Mean ± SD	CI^b^	Mean ± SD	CI^b^	
CC-RA	96 ± 21	83–110	98 ± 13	89–106	*p* = 0.67
CC-D (%)	48 ± 42	21–74	70 ± 31	50–90	*p* = 0.02 [ES 0.48]^a^
CC-RE (%)	33 ± 40	8–58	41 ± 34	19–63	*p* = 0.21
CC-HP (%)	53 ± 49	22–84	54 ± 49	23–85	*p* = 0.89

*CI* confidence interval, *CPR* cardiopulmonary resuscitation, *SD* standard deviation, *SGs* smart glasses, *CC-RA* chest compression (CC) rate, *CC-D* CC depth (50–60 mm) in percentage, *CC-RE* CC with full chest recoil in percentage, *CC-HP* CC with correct hands placement in percentage

^a^Small effect size with Rosenthal test

^b^95% CI

## Discussion

The aim of this study was to evaluate the feasibility of SGs as a means of support between an emergency dispatcher and a bystander during BLS in a special resuscitation setting, such as a small fishing boat during sea transport.

The main findings were: (a) The use of SGs was feasible in all tests and helped the bystanders to correctly follow all BLS steps in a cardiac-arrest-simulated scenario; (b) SGs were especially useful during the ABC approach and the AED response, but to a lesser extent in CPR-quality markers; (c) the final evaluation of the items corrected by the dispatcher had a high agreement with the assessment of the items by the on-scene instructor, suggesting a high level of reliability.

Witnesses have a fundamental role in the treatment of OHCA [8] but they do not always take the initiative, often failing to recognize cardiac arrest or simply feeling unable to act [9]. However, with the development of telecommunications [10], the identification and response to OHCA have improved with the emergence of the emergency dispatcher [11, 12]. The evolution of 4G and 5G wireless systems [9] is leading to a transition from telephone dispatchers to a new form of dispatcher based on real-time video streaming [11, 13], although there is still a lack of evidence in this regard [10]. Scientific literature has identified limitations related to the number of witnesses required for the video call (at least two) [11, 13], the difficulty of communication between the dispatcher and the bystander [14, 15] or even the position of the camera [16]. A large number of these limitations may be due to the type of device most common in this type of communication: the smartphone [9–11, 15, 17]. However, SGs can circumvent these limitations, as they connect the responder with the dispatcher first, without the need for additional witnesses. In addition, they enable hands-free use and the camera position is right above the victim.

One of the key strengths of livestreaming is that the dispatcher can see what is happening and make decisions about it. In a studio in Copenhagen called Good Sam, a telephone dispatcher was offered first and then a video call dispatcher if there were two witnesses. The live transmission was successful in 82% of the calls and the condition of the patients changed in 51%, resulting in a change in the emergency of 27.5% after receiving the video support [11]. In this study, communication could be established in all tests and the dispatcher was able to guide or correct the bystander´s maneuvers in 72% of the ABC stages and improve CC depth.

The most relevant and pertinent aspects in cardiac arrest is early defibrillation and early CPR [1, 17]. The fishermen in this study took 2.5 min to deliver the first shock and 14 s longer to initiate CPR. Although the aim is to do this as soon as possible, they would probably take longer or fail without the help of the dispatcher, who could see that no fisherman approached the AED on their own (without the help of the dispatcher through the SG) and that on numerous occasions, the fishermen did not place the AED patches correctly. In real life, defibrillation delays of 2 min or more are not uncommon even with medical staff and AED in situ, as was observed in the cardiac arrest during the broadcast of the Euro 2021 international soccer competition [18].

The study by Bolle et al. with young video-guided students showed that it took approximately 1.5 min from the start of the test to the first compression [15], but it should be noted that this was a controlled environment, very different from that of fishermen sailing during their intervention.

Emergency dispatchers are trained to provide CPR instructions following a predefined protocol [19]. Ecker et al. found improved performance when bystanders performed CPR with video assistance vs. only with call assistance. Significant differences in the depth of compression were observed but especially in the placement of hands in the correct compression site [9].

However, there were no major differences in the comparison with audio dispatcher assistance. In this study, the comparison of the 1st minute without dispatcher feedback vs. the 2nd minute, the emergency dispatcher provided ongoing feedback through the SGs. A significant improvement in the CC depth was achieved with 70% quality in CC-D, but not so in CC-HP which barely surpassed 50% in quality.

Then how was it possible that the bystanders’ depth results improved with SGs, while there was no modification in the position of the hands? In the authors’ opinion, the zenithal view can provide a depth perspective since the sinking of the chest is noticeable, but the contact surface of the CC cannot be visualized. The dispatcher cannot tell whether the bystander is supporting the whole hand or just the heel. In addition, the CC reference is lost several times because during CPR, the anatomical position with the head projected beyond the arms causes the camera view to be lost in this maneuver.

This circumstance could be corrected by modifying the protocols of the dispatcher for handling video calls [15] and with technical adaptations such as a wide-angle lens. The study of the camera position is a relevant issue and the study by Wetsch et al. with smartphones placed on a tripod suggests that the best location is the side position, since it offers better error detection, but only for the CPR and not for the BLS sequence [16]. A novelty of this study is the zenith and dynamic position of the camera in the SGs, with the bystander’s hands free, which allowed the dispatcher to perform a good evaluation of the ABC approach albeit with errors in the HP. This error caused by the device could be seen in the reliability analysis through the on-scene instructor analysis. In all other steps, there was almost complete agreement between the two evaluations.

### Implications for practice

This study has served to test a commercial model of SGs with features common to most of the devices available on the market, but not specifically designed to guide BLS. The major strength is the possibility for a single hands-free witness to have quality two-way communication in a challenging setting such as an aquatic environment, which is a non-controllable scenario.

Another strength is the bone conduction hearing mechanism, which makes it easy to receive the dispatcher's indications even in spite of wind or engine noise. SGs are currently an affordable product and their price is similar to that of high-end smartphones. On the other hand, they also have important limitations, such as camera angle range or switch on. In our study, the device was switched on, but the start-up time is approximately 40 s. Another limiting aspect is bystanders with optical problem (such as myopia) as he or she would have difficulty viewing the display projected on the optics. During testing, heating of the device was noticed which should be addressed by the manufacturers. Prolonged use could cause discomfort for the rescuer or failures in the device itself (i.e., automatic switch off). To improve the procedure, the dispatcher could ask the bystander, wearing the SG, to look at the position of his/her own hands during the first CCs.

### Limitations of this study

This study was proposed as a pilot feasibility study to test the usability of the glasses in a special setting with fishermen. For this reason, the sample size was small. Therefore, the study was not powered to investigate possible differences in CPR-quality markers. The results may differ with larger samples, in other settings, and with other conditions. Connectivity at the study location was advanced and in other regions that might be a major limitation. This was a simulation study, so applicability in real situations should be carefully evaluated before implementation.

## Conclusion

The use of SGs in aquatic settings seems feasible if the right wireless connectivity conditions are available. Communication between the emergency dispatcher and the witness is seamless and is especially helpful during the dispatch of the ABC approach and AED use. The small sample size did not allow to investigate significant differences in CPR-quality markers. We consider that these devices have great potential for communication between dispatchers and laypersons but need improvement to be used in real emergencies.

## Supplementary Information

Below is the link to the electronic supplementary material.

Supplementary file1 (MP4 28346 KB)

## Data Availability

The datasets generated during and/or analysed during the current study are available from the corresponding author on reasonable request.

## Abbreviations

AED: Automated external defibrillator
ABC: Airway, breathing and circulation
BLS: Basic life support
CC: Chest compressions
CPR: Cardiopulmonary resuscitation
ERC: European resuscitation council
EMS: Emergency medical services
OHCA: Out-of-hospital cardiac arrest
SGs: Smart glasses
